# Heat and Mass Transfer in Porous Materials

**DOI:** 10.3390/ma16165591

**Published:** 2023-08-12

**Authors:** Anatoliy Pavlenko

**Affiliations:** Department of Building Physics and Renewable Energy, Kielce University of Technology, Aleja Tysiąclecia Państwa Polskiego, 7, 25-314 Kielce, Poland; apavlenko@tu.kielce.pl

## 1. Introduction

Currently, porous materials (PM) are actively used in many fields of science and technology, and the processes of heat and mass transfer in porous materials underlie a wide variety of industrial technologies. For example, porous materials are used in power plants, heat and mass transfer devices, heat pumps, etc. [1,2,3,4,5,6,7]. Given that almost all technologies are associated with porous media and heat- and mass-transfer processes in them both in technological processes and in nature, the area of scientific applications of PM is practically unlimited. Porous media play an important role in a wide range of scientific and engineering problems. Therefore, the problems of their application are associated with the solution of multiscale processes that combine the transfer of mass, momentum, and energy [8,9].

Heat and mass transfer processes in PM are part of our daily experience, and this general process is central to many environmental and engineering applications, from the evaporation of moisture from the soil to the drying of various products and building materials. The intensity of heat and mass transfer in porous media can exhibit complex dynamics, reflecting internal transport mechanisms and the motion of the phase transition front, which critically affect the distribution of surface energy [10,11,12,13,14,15,16].

One of the key thermophysical properties of porous media is effective heat and mass conduction. Identification and quantification of morphological features that correlate with thermal conductivity are vital to understanding the mechanism of thermal conductivity in porous media [17].

Thus, heat and mass transfer in porous media has been an important research topic for many decades because of its applicability. Despite numerous studies over more than a century, there are many new discoveries that are still improving the basic understanding of the subject.

A special issue of this journal is devoted to the topical scientific problem of studying the processes of interconnected heat and mass transfer in porous media. This problem is one of the complex and important fundamental areas of modern science and is of significant applied importance. The results of studies of heat and mass transfer in porous materials can be used to intensify heat transfer in various power plants to increase their energy efficiency.

The purpose of this special issue is to showcase the latest developments in PM heat transfer and mass transfer technologies that contribute to sustainable development. Although significant attention has been paid to these issues, there remains a constant demand for innovative solutions to address a wide range of problems in this area. Therefore, this special issue attempts to highlight and propose new solutions to these problems [18,19,20,21,22,23,24,25,26,27]. These works develop a basic understanding of the scientific problems of heat and mass transfer in porous materials. Thematically, three areas can be distinguished, covered by the studies of the articles published in this special issue: mass transfer in porous media, methods for forming a porous medium, conjugate heat, and mass transfer.

## 2. A Review of the Contributions in This Issue

### 2.1. Mass Transfer in Porous Media

Recently, there has been increasing interest in the creation of new highly porous nanocarbons, such as fullerenes, nanotubes, and nanofibers, both for basic research and for potential applications. The carbon material is characterized by increased hierarchical nanoporosity, good electrical conductivity and high thermal stability.

In the article by the authors Katarzyna Skrzypczyńska et al. [18] developed the ideas outlined in [28,29,30,31,32,33,34,35,36], studied the properties of carbon materials, showed and confirmed that the products obtained in the magnesium thermal process contain carbon, graphene material with certain micro and mesopores. Compared to other works on the preparation of such specific carbon materials by combustion synthesis, the PMs analyzed in this work were obtained using organic as well as inorganic salts containing carbon in their anions, which gives them specific features.

In [19], Kuśmierek K. and others developed the topic of research on highly porous carbon materials. The authors focused on carbon materials with different porosities: single-walled carbon nanotubes, heat-treated activated carbon, and reduced graphene oxide for studying adsorption processes. The dependence of the adsorption and electrochemical properties of these materials on their porosity has been established.

The authors of Maier L. et al. in their work [20] develop the idea of correlations between the permeability of fibrous materials according to their porosity for ordered fibrous media to estimate the effective diffusion and permeability of filaments depending on the porosity and diameter of the fibre, which take into account random ordering.

### 2.2. Methods for the Formation of a Porous Medium

In microporous and nanoporous materials, their technological characteristics depend on the distribution of pores in size, volume, and shape. This has become a hot area of research for the development of materials with precisely controlled pores and volume distribution. Recent studies [37,38,39,40,41,42,43,44,45,46,47] have focused more on precise control of pore shapes, sizes, and volumes to produce high-performance porous materials. The formation of pores in a material can introduce striking features into the material due to its specific surface-to-volume ratio. Their outstanding performance and nanoporous structures have made these materials valuable in the fields of environmental remediation, adsorption, catalysis, energy conversion, purification, medicine and ecology.

They can be divided into three main groups: carbon nanoporous materials, which we mentioned above; organic polymer nanoporous materials and inorganic PM.

Inorganic nanoporous materials include, in particular, zeolites, which is the method of obtaining which is presented in this special issue by the authors of Tontisirin, S. et al. in [21].

In [22], the results of studies of the capillary effect in porous structures with different pore sizes are presented in order to develop passive cooling systems based on the mechanisms of the liquid-vapor phase transition to remove large heat fluxes. The high intensity and density of energy flow in some industries require efficient and reliable heat transfer. In such technologies, heat pipes are actively used since they have the ability to transfer heat efficiently. This topic is developed by the authors of whose article [23], the results are related to the production of heat pipes. A new approach in their products is the use of high-tech additive manufacturing technologies, in which the most complex geometries are made layer by layer directly from a digital file. This technology produces efficient homogeneous structures with the desired porosity, uniform pore size, permeability, thickness, and uniform pore distribution.

In [24], the direction of influence of the flow on perforated plates on their aerodynamic characteristics is considered, and the effect of laser technology on the flow around the perforated plates is considered. The aerodynamic characteristics for different plates with different perforations, diameters, and lengths of microchannels are presented.

### 2.3. Conjugate Heat and Mass Transfer

The phenomena of heat and mass transfer in porous media play an important role in the process of coupled heat and mass transfer. They are found in many applications, in industrial applications, and in natural processes. A typical case for these applications is the condensation of steam in building walls, in which the absorption of moisture has a very detrimental effect on the thermal and mechanical properties of the materials. It is known that when a PM is in contact with warm, moist air on one side and with a cold wall on the other side, condensation can occur in the PM at certain temperatures and humidity, which will affect the thermal insulation and mechanical properties of the structure [25]. Despite the fact that mathematical modelling of heat and mass transfer in porous media has been well studied [48,49,50,51,52,53,54,55,56,57,58,59,60,61], the mathematical approaches and assumptions used in many cases give only approximate predictions of the dynamics of this phenomenon.

The research work [26] presents modelling of the dynamics of a vapour-gas mixture and heat and mass transfer in the capillary structure of a porous medium. At the heart of the approach implemented in this article, the porous structure is represented by a system of linear microchannels in a three-dimensional coordinate system. The area of the channels is modelled by a set of cubic elements with a certain humidity, moisture content, pressure, and temperature. Such a modelling scheme includes a certain number of parameters, thermophysical properties, and characteristics of the porous material, depending on the moisture content. The authors of the article directly considered the effects of heat and mass transfer in the structure of the material and phase transition-evaporation or condensation in the elements of the porous structure.

Another important class of heat and mass transfer problems in porous media is determined by physical processes in the environment, for example, in soil, when low-grade heat is used to operate heat pumps [62,63,64].

Compared to heat transfer, moisture migration in soils is more complex and includes molecular, capillary, and osmotic diffusion. Thus, almost all parameters of the microstructure of a porous medium will affect its heat or mass flow. An article by Borys Basok, Borys Davydenko, Hanna Koshlak, and Volodymyr Novikov [27] is devoted to solving this problem. In this paper, using numerical simulation methods, a mathematical model of heat transfer processes in a porous soil mass in the vicinity of a Y-shaped vertical heat exchanger is described. The purpose of these studies is to determine the influence of the filtration properties of the soil, as a porous medium, on the performance characteristics of soil heat exchangers. Based on the results of the numerical solution of the system of equations of hydrodynamics and heat transfer in a porous medium filled with water and air, the characteristics of a free-convective fluid flow in a soil mass in the presence of a vertical heat exchanger are determined.

## 3. Conclusions

The section “Mass transfer in porous media” of the special issue is devoted to fundamental research and the development of new materials for various industrial applications. The category “Methods for the formation of a porous medium” considers new approaches and technologies for the formation of a porous structure with certain properties that determine the effectiveness of the use of these materials in various technologies in the fields of environmental restoration, adsorption, catalysis, energy conversion, purification, medicine, etc. The results of the research are presented. effects in porous structures with different pore sizes to develop passive cooling systems, research into the production of heat pipes, and additive manufacturing technologies for materials with complex geometries with the desired porosity, uniform pore sizes, permeability, thickness, and uniform distribution of pores.

The category “Conjugate heat and mass transfer” explores the dynamics of the vapour-gas mixture and heat and mass transfer in the capillary structure of a porous medium.

The articles in this special issue offer innovative solutions to the problems of heat and mass transfer in porous materials, promoting sustainability and energy efficiency. These solutions can be applied in a variety of settings to improve energy management practices.
